# Management of COVID-19 infection in patients undergoing hemodialysis

**DOI:** 10.11604/pamj.2021.38.13.27151

**Published:** 2021-01-06

**Authors:** Boushab Mohamed Boushab, Noukhoum Koné, Sidi El-Wafi Ould Baba, Mohamed Jeddou Ould Mohamed El Mokhtar Bellattv, Mohamed Moustava Ould Ahmed, Houssein Ould Habiboullah, El-Vak Ould Ahmed Baba, Oumar Ould Sidi Mohamed, Leonardo Kishi Basco

**Affiliations:** 1 Department of Internal Medicine and Infectious Diseases, Kiffa Regional Hospital, Assaba, Mauritania,; 2 Department of Medicine and Medical Specialty, Faculty of Medicine, Université de Nouakchott Al Aasriya, Nouakchott, Mauritania,; 3 Department of Neurosurgery, Kiffa Regional Hospital, Assaba, Mauritania,; 4 Department of Hemodialysis, Kiffa Regional Hospital, Assaba, Mauritania,; 5 Department of Emergency Medicine, Kiffa Regional Hospital, Assaba, Mauritania,; 6 Direction Régionale Action Sanitaire de l´Assaba, Assaba, Mauritania,; 7 Aix-Marseille Univ, IRD, AP-HM, SSA, VITROME, Marseille, France,; 8 IHU-Méditerranée Infection, Marseille, France

**Keywords:** COVID-19, epidemic, dialysis patients, Mauritania

## Abstract

Coronavirus disease 2019 (COVID-19), a pandemic affecting the world's population, is particularly threatening to dialysis patients. The purpose of this study was to propose recommendations for prevention and containment of COVID-19 in hemodialysis center situated in a regional hospital in Mauritania. Both COVID-19-infected (n = 11) and -uninfected (n = 16) dialysis patients were hospitalized up to three weeks to improve patient management. All COVID-19-infected patients were cured. Patient care and hygiene in a safe environment are the key factors for a favorable outcome, even in resource-limited settings.

## Introduction

Patients with end stage renal disease on dialysis are particularly vulnerable to coronavirus disease 2019 (COVID-19) [1]. Many of these patients require regular weekly sessions of hemodialysis in a hospital, and special care and protective measures are needed to create a safe environment in hospitals for these patients [2]. Data on hospital management of COVID-19 infected patients with end-stage renal disease undergoing hemodialysis are limited [3,4]. We report here our experience of COVID-19 management in dialysis patients in a resource-limited setting in Mauritania.

## Methods

The study was conducted at Kiffa regional hospital, a regional reference health center located in Kiffa, the second largest city in Mauritania located about 600km from Nouakchott, the capital city, in Assaba region, between May 15 and June 15, 2020. All adult dialysis patients were enrolled after written informed consent and screened for COVID-19. Oropharyngeal swabs were collected and sent to the Mauritanian National Institute for Research in Public Health in Nouakchott for COVID-19 diagnosis by reverse transcriptase PCR (LightMix® modular SARS-CoV-2 (COVID19) RdRP, F. Hoffmann-La Roche, Basel, Switzerland). Clinical and epidemiological data, including the probable place of exposure to COVID-19 and contact with confirmed or suspected cases of COVID-19, were recorded. COVID-19-positive dialysis patients were treated with oral hydroxychloroquine-azithromycin combination only if the patients gave their informed consent [5]. Patients with past history of allergic reactions to hydroxychloroquine, chloroquine, or azithromycin, those with any cardiovascular disease, patients under treatment with drugs known to prolong QT interval, and patients with severe acute respiratory distress were excluded from this treatment regimen. Due to limited financial resources of dialysis patients, electrocardiogram and radiographical examination were not performed. Follow-up diagnostic PCRs for COVID-19 were performed on day 7, day 10, day 14, and then weekly until PCR became negative. The criteria for recovery from COVID-19 infection and hospital discharge were as follows: (i) normal respiratory rate (< 20 cycles per minute), (ii) absence of fever, (iii) absence of need for oxygen therapy on day 6, and (iv) two negative PCR tests performed with an interval of 48 hours. The present clinical protocol was approved by the Mauritanian Ministry of Health.

## Results

Among 27 dialysis patients hospitalized during the study period, 11 (41%) were tested positive for COVID-19 on admission. The median age was 50.5 ± 14.0 years (range: 22 to 70 years), and the male-to-female sex ratio was 0.57. Five of 11 (45%) COVID-19 positive patients were asymptomatic. Flu-like signs and symptoms were present in 6 (55%) patients at the time of hospital admission, including fever (n = 6), minor nose and throat irritation (n = 3), cough (n = 2), headache (n = 2), myalgia (n = 1), and hiccups (n = 1). Among these 6 patients, the delay between the onset of symptoms to hospitalization was, on the average, 4.4 days (range: 3 to 6 days). None required oxygen therapy on admission. Three of 6 symptomatic COVID-19-positive patients gave their consent for hydroxychloroquine-azithromycin therapy. Two of them refused to pursue the treatment on day 1 due to abdominal pain and nausea, which may or may not be attributable to the treatment. Symptomatic treatment (paracetamol, ferrous sulfate) was given to all dialysis patients, as required. All dialysis patients were also under low-dose aspirin (100 mg/day), except on the day of dialysis when heparin was administered. Patients requiring antihypertensive treatment were given their usual medications. None of the included patients with confirmed COVID-19 required ventilator support during hospitalization. Dialysis sessions were maintained as part of a routine protocol. Of 11 COVID-19-positive patients, 9 had their first negative PCR on day 10, followed by a second negative PCR on day 12. These patients were considered to be cured and discharged on day 15. Two other patients had to be confined until day 21 before hospital discharge. The mean duration of confinement at the hospital was 16.1 ± 2.4 days, with a range from 15 to 21 days. There was no death or renal, pulmonary, or cardiovascular complication among COVID-19-positive dialysis patients. Upon discharge from hospital, patients who were COVID-19 positive were strongly advised to stay confined in their homes for an additional 14 days.

## Discussion

Chronic kidney disease is one of the co-morbidities that may render an affected patient more vulnerable to complications associated with COVID-19, and in this context dialysis centers may become a potential source of spread of this virus [2,4,6]. In our study, the mean period of incubation, defined as the interval between the first contact with suspected or confirmed case of COVID-19 and the onset of COVID-19 associated signs and symptoms, was 4.4 days, which is in agreement with the incubation period estimated in other studies [7,8]. In some COVID-19 infected patients, the initial phase of viral invasion is followed by an aggravation of respiratory symptoms and inflammatory syndrome, referred to as “cytokine storm,” generally eight to ten days after the onset of symptoms [9]. Based on the hypothesis that inflammation and occlusion of pulmonary arterioles may result in pulmonary lesions and hypoxia, low-dose aspirin was administered to our dialysis patients, except on the day of dialysis. None of our COVID-19-positive patients progressed to this potentially fatal second phase, which explains the favorable outcome in all our patients. There is currently no specific treatment or vaccine. However, several compounds, such as hydroxychloroquine and remdesivir, have been proposed as possible leads [5,10]. In our study, only one patient completed the full hydroxychloroquine-azithromycin treatment and, like other patients who did not take this treatment, was cured. We deduce that hydroxychloroquine-azithromycin probably did not contribute to the favorable clinical outcome.

Since home dialysis is beyond the financial means of most Mauritanian patients, all COVID-19-infected dialysis patients were hospitalized and confined in our hospital ward for at least 14 days. Hospitalization limited the movement of patients between their home and the hemodialysis unit and reduced the risk of spread of the virus. Hospitalization also allowed patients to continue their dialysis sessions and follow-up without coming in contact with persons outside the hospital. A maximum of two patients were dialyzed at the same time in the dialysis station, with a distance of at least two meters separating them. The treatment areas and rooms were air conditioned and ventilated to remove aerosol particles and droplets in the air. Each patient was confined in an individual room and was allowed to leave the assigned room only for dialysis sessions. Body temperature was taken regularly. All personnel involved in the care of COVID-19 infected patients were provided with complete protection, and hand hygiene was strictly implemented. These measures were effective in protecting both COVID-19-infected and -uninfected dialysis patients and their accompanying family members in our hospital. Moreover, upon hospital discharge, the patients were instructed to stay home and avoid public transportation, contact with other persons, and group events.

## Conclusion

Dialysis patients are particularly vulnerable to COVID-19. Patient management is mainly supportive and should be performed according to strict protocols to minimize the risk not only for dialysis patients themselves, but also for other patients and the hospital staff. Prevention, screening, and temporary isolation have proven to be effective in our hospital settings despite limited resources. In the absence of a specific curative treatment and effective vaccine, prevention remains the best means to fight against this potentially fatal disease.

### What is known about this topic

- Dialysis is a therapeutic medical act;
- Each dialysis patient is at risk of transmissible infections if the measures are not secure;
- Morbidity and mortality resulting from covid-19 have serious consequences for patients, communities and society in general.

### What this study adds

- To our knowledge, this is the first study in the country to evaluate COVID-19 management among dialysis patients;
- Limit the spread of the virus;
- Strengthen barrier measures among dialysis patients.
